# Akt1-mediated CPR cooling protection targets regulators of metabolism, inflammation and contractile function in mouse cardiac arrest

**DOI:** 10.1371/journal.pone.0220604

**Published:** 2019-08-09

**Authors:** Jing Li, Xiangdong Zhu, Huashan Wang, Chunpei Lee, Sy-Jou Chen, Yuanyu Qian, Mei Han, Ryan Bunney, David G. Beiser, Terry L. Vanden Hoek

**Affiliations:** 1 Center for Advanced Resuscitation Medicine, Center for Cardiovascular Research, and Department of Emergency Medicine, University of Illinois Hospital & Health Sciences System, Chicago, Illinois, United States of America; 2 Department of Emergency Medicine, Tri-Service General Hospital, National Defense Medical Center, Taipei, Taiwan; 3 Emergency Department, Chinese PLA General Hospital, Beijing, China; 4 Section of Emergency Medicine, Department of Medicine, University of Chicago, Chicago, Illinois, United States of America; Virginia Commonwealth University, UNITED STATES

## Abstract

Therapeutic hypothermia initiated during cardiopulmonary resuscitation (CPR) in pre-clinical studies appears to be highly protective against sudden cardiac arrest injury. Given the challenges to implementing CPR cooling clinically, insights into its critical mechanisms of protection could guide development of new CPR drugs that mimic hypothermia effects without the need for physical cooling. Here, we used Akt1-deficient mice that lose CPR hypothermia protection to identify hypothermia targets. Adult female C57BL/6 mice (Akt1^+/+^ and Akt1^+/-^) underwent 8 min of KCl-induced asystolic arrest and were randomized to receive hypothermia (30 ± 0.5°C) or normothermia. Hypothermia was initiated during CPR and extended for 1 h after resuscitation. Neurologically scored survival was measured at 72 h. Other outcomes included mean arterial pressure and target measures in heart and brain related to contractile function, glucose utilization and inflammation. Compared to northothermia, hypothermia improved both 2h mean arterial pressure and 72h neurologically intact survival in Akt1^+/+^ mice but not in Akt1^+/-^ mice. In Akt1^+/+^ mice, hypothermia increased Akt and GSK3β phosphorylation, pyruvate dehydrogenase activation, and NAD^+^ and ATP production while decreasing IκBα degradation and NF-κB activity in both heart and brain at 30 min after CPR. It also increased phospholamban phosphorylation in heart tissue. Further, hypothermia reduced metabolic and inflammatory blood markers lactate and Pre-B cell Colony Enhancing Factor. Despite hypothermia treatment, all these effects were reversed in Akt1^+/-^ mice. Taken together, drugs that target Akt1 and its effectors may have the potential to mimic hypothermia-like protection to improve sudden cardiac arrest survival when administered during CPR.

## Introduction

Out-of-hospital sudden cardiac arrest (SCA) affects about 1000 people each day in the United States with an overall survival rate of about 7% [1]. Significant mortality is due to a post-SCA syndrome consisting of myocardial stunning and hypotension, underlying metabolic injury demonstrated by decreased glucose utilization and lactic acidosis, and sepsis-like systemic inflammation. These hemodynamic, metabolic and inflammatory aspects of the post-SCA syndrome contribute to neurologic injury and death [2–4]. Stunned myocardium is an early hallmark of SCA injury, resulting from inactivation of pyruvate dehydrogenase (PDH) as well as alteration of calcium homeostasis and contractile protein structure [5]. Heart and brain recovery after SCA are likely interrelated both by common mechanisms of injury and recovery (e.g. PDH inhibition versus activation) [6, 7] as well as the link between improved cardiac function and increased tissue perfusion by 6 h after CPR with 72 h neurological recovery [8].

While therapeutic hypothermia/cooling (TH) induced within hours after SCA is currently used clinically to protect against neurologic injury. Active cooling initiated more rapidly after return of spontaneous circulation (ROSC) has been shown to confer cardioprotection in various animal models [9, 10] and in human studies of cooling [11]. We and others have shown that TH induced earlier during CPR prior to ROSC is highly protective and potent enough to require only an hour of cooling to provide significant benefit [10, 12, 13] compared to 12–24 h of cooling required when initiated after ROSC. However, TH started at CPR is difficult to implement clinically.

Here, we used Akt1-deficient mice that have been previously shown to lose TH cardioprotection compared to wild type mice [14] to identify an Akt1-related “molecular signature” of TH protection. Such a signature would be helpful to guide therapeutics designed to transiently activate Akt during CPR without the need for physical cooling. Akt consists of three isoforms, Akt1, Akt2 and Akt3. Akt1 predominantly resides in heart, brain (i.e. glial support cells) and lung, whereas Akt2 is largely expressed in skeletal muscle, embryonic brown fat, heart, liver and kidney, and Akt3 is abundant in the brain and kidney [15, 16]. We have recently demonstrated that a biological fusion protein inhibitor (TAT-ΔPTENp85) and a small chemical inhibitor (VO-OHpic) of phosphatase PTEN, that both negatively regulates Akt activity, improved survival from ischemic/reperfusion injury in a mouse cardiomyocyte I/R model and a mouse SCA model, respectively [17, 18]. The prior work has identified that Akt1 is a critical regulator of TH-mediated hemodynamic recovery and 4 hour survival after SCA. The present work further studied Akt1-related loss of CPR TH protection by studying mechanistic actions of Akt1. We hypothesized that Akt1 is critical to CPR TH-mediated long-term neurologically intact survival through its targets regulating contractile function, metabolic recovery, and inflammatory responses. This work identifies Akt1 targets involved in these aspects during early resuscitation in multiple organs including heart, brain and blood of SCA mice.

## Materials and methods

### Mouse model of sudden cardiac arrest and TH treatment

All procedures were performed under a protocol approved by the Institutional Animal Care and Use Committee of the University of Illinois at Chicago and University of Chicago. The procedures have been previously described [14, 18]. In brief, 4–8 months old (weight between 23–30 g) wild type (Akt1^+/+^) and Akt heterozygote (Akt1^+/-^) female C57BL/6 were used. Mice were anesthetized with 100 mg/kg ketamine and 10 mg/kg xylazine. The trachea, carotid artery and internal jugular vein were surgically exposed via a midline ventral neck incision. Following exposure, the trachea was then intubated with a 20G angiocath under direct visualization and a polyethylene (PE-10) catheter was inserted into the carotid artery for blood pressure monitoring. A second PE-10 catheter was inserted into a jugular vein. ECG needle probes were then inserted subcutaneously for cardiac monitoring. Rectal temperature was monitored and maintained at 37 ± 0.5°C throughout the surgical preparation. Standard ventilation settings for most experiments include the respiratory rate of 110, tidal volume of 12.5 μl/g animal weight, FiO_2_ of 1.0 and positive end-expiratory pressure of 2 cm H_2_O. Following 20 min stabilization with MAP > 80 mmHg, and a partial pressure of end-tidal CO_2_ (P_ETCO2_) > 35 mmHg, asystolic cardiac arrest was then induced by intravenous administration of 0.08 mg/g potassium chloride solution. After 6 min of cardiac arrest induction, mice were randomized to either continued normothermia (NT, 37 ± 0.5°C in Akt1^+/+^mice) or TH (30 ± 0.5°C in both Akt1^+/+^ and Akt1^+/-^ mice). TH was induced using a small bag containing ice-cold water on the chest after 6 min arrest (the last 2 min of 8 min arrest). The temperature of 30 ± 0.5°C was achieved 2–3 minutes after the application of ice-cold water and demonstrated a remarkable protection against cardiac arrest in mouse [10]. Mice were resuscitated at the end of the 8 min arrest. Mechanical ventilation was re-started and chest compressions at a rate of 300–400 compressions/min are initiated by finger application for up to 5 min. Animals received a bolus of epinephrine (0.05 mg/kg) at 90 second of resuscitation period. At 60 min post-ROSC, TH animals were rewarmed over 20 min to 37 ± 0.5°C. Sham mice underwent instrumentation but not cardiac arrest. In this study, Sixty-five mice were used. Among them, thirty mice were euthanized including one mouse who presented a sinus rhythm after KCl injection. Thirty-five mice died before the end of the protocol including four mice who died from the technical failure. The primary cause of the death was cardiac arrest injury.

### Survival and non-survival studies

Mice were randomized to receive TH or NT (n = 10 each group) and used for survival studies. Survival study animals remained on mechanical ventilation and under hemodynamic monitoring for 2 hours until they showed evidence of spontaneous breathing along with adequate mean arterial pressures (> 55 mmHg). At this time, animals were disconnected from the ventilator for a breathing trial. Once it was determined that the animal was able to breathe on its own, the animal was extubated and all vascular access devices were removed, vessels ligated and surgical wounds sutured. Prior to awakening, animals received a dose of buprenorphine (0.1 mg/kg) for pain control. The animals were placed in a protected recovery area and visually monitored for 2 hours before animals were returned and individually housed in the animal facility for observation twice a day up to 72 hours. The neurological function of survived mice was evaluated with an established scoring system including level of consciousness, corneal reflex, respiration, righting reflex, coordination and movement/activity. The neurological score was assigned according to the prior studies [18, 19]. The scores range from 0 (death) to 12 (normal neurological function). The animals that survived to the end of the protocol were euthanized by CO_2_ inhalation and cervical dislocation. For non-survival study, ROSC animals were continuously observed for up to 30 min. Animals were sacrificed under anesthesia for tissue harvest at either the end of protocol or when the MAP fell below 30 mmHg for over 5 min.

### Western blot analysis

Heart and brain tissues were collected at 30 min post-ROSC (R30) from four groups of mice (sham, NT and TH in Akt^+/+^ and TH in Akt1^+/-^, n = 5 in each group) and lysed for protein analysis by Western blot as previously described [20]. In brief, proteins in the tissue samples were separated by 7.5–12% SDS-page gels and transferred to PVDF membranes. After blocking in 5% milk/TBST buffer for 1 hour, the membranes were incubated with various antibodies diluted in 1% BSA/TBST buffer for either 1 hour at room temperature or overnight at 4°C, then exposed to HRP-linked goat anti-rabbit or HRP-linked goat anti-mouse and finally exposed to SuperSignal (Thermol Scientific, Rockford, IL) for visualization. Protein phosphorylation and expression were detected by antibodies against phosphorylation of Akt Thr308 (Cell Signaling Technology #9275, Danvers, MA), Akt Ser473 (Cell Signaling #4058), GSK3β Ser9 (Cell Signaling #9336), phospholamban Ser16/Thr17 (Cell Signaling #8496) and pyruvate dehydrogenase (PDH) E1-alpha subunit p-Ser293 (Novus Biologicals, NB110-93479, Littleton, CO), Akt1 (Cell Signaling #2967), Akt (Cell Signaling #9272), IκBα (Cell Signaling #9242), α-tubulin (used as a loading control in heart, NeoMarkers, MS581-P, Fremont, CA) and β-actin (used as a loading control in brain, Sigma-Aldrich, A5441 St. Louis, MO). For visualization, HRP-linked goat anti-rabbit (Cell Signaling #7074) or HRP-linked goat anti-mouse (Cell Signaling #7076) were used at 1:2000 dilution. Quantitative results were obtained via densitometry (NIH ImageJ version 1.42, National Institutes of Health, Bethesda).

### NF-κB activity assay

Nuclear extracts were isolated from heart and brain tissues collected at R30 using a commercially available nuclear extract kit (EMD #2900, Billerica, MA). The DNA binding activity of NF-κB (p50/p65) in the nuclear fraction was determined using an ELISA-based nonradioactive NF-κB p50/p65 transcription factor assay kit according to the manufacturer's protocol (Abcam #ab133112, Cambridge, MA).

### NAD^+^ and ATP measurement

NAD^+^ and ATP concentrations of heart and brain at R30 were measured with a colormetric NAD^+^ assay kit (Cayman Chemical #600480, Ann Arbor, MI) and a colometric ATP assay kit (Abcam #ab83355, Cambridge, MA), respectively, by following manufacturer’s instructions.

### Measurement of plasma lactate and Pre-B Cell Colony Enhancing Factor (PBEF)

Mice used for tissue collection at R30 were also used to measure concurrent plasma L-lactate using a colorimetric assay kit (Abcam #ab65331, Cambridge, MA) and a pro-inflammatory plasma cytokine PBEF using an ELISA kit (MBL International Corporation #CY8065V2, Woburn, MA). Blood samples (about 200–500 μl per mouse) were collected into heparin-coated tubes from artery line, then centrifuged within 30 minutes of collection at 2000 g for 10 minutes. Then plasma samples were transferred to new tubes and stored at 80°C. Plasma samples were used as a 1:4 dilution and the assay was performed in accordance with manufacturer’s protocols.

### Statistical analysis

All statistical analysis was performed using OriginPro 8.5 (OriginLab, Northampton, MA). Results were expressed as mean ± SEM. For comparison among the different treatment groups, one-way ANOVA was used with *post-hoc* examination by Tukey’s test. For survival analysis log-rank (Mantel-Cox) test using Kaplan-Meier curves was conducted. For subgroup comparisons, t-test was used for mean arterial pressure analysis.A value of *p* < 0.05 was considered statistically significant.

## Results

### Akt1 deficiency abolished CPR TH benefits on cardiac and brain function recovery and survival

**Fig 1:**
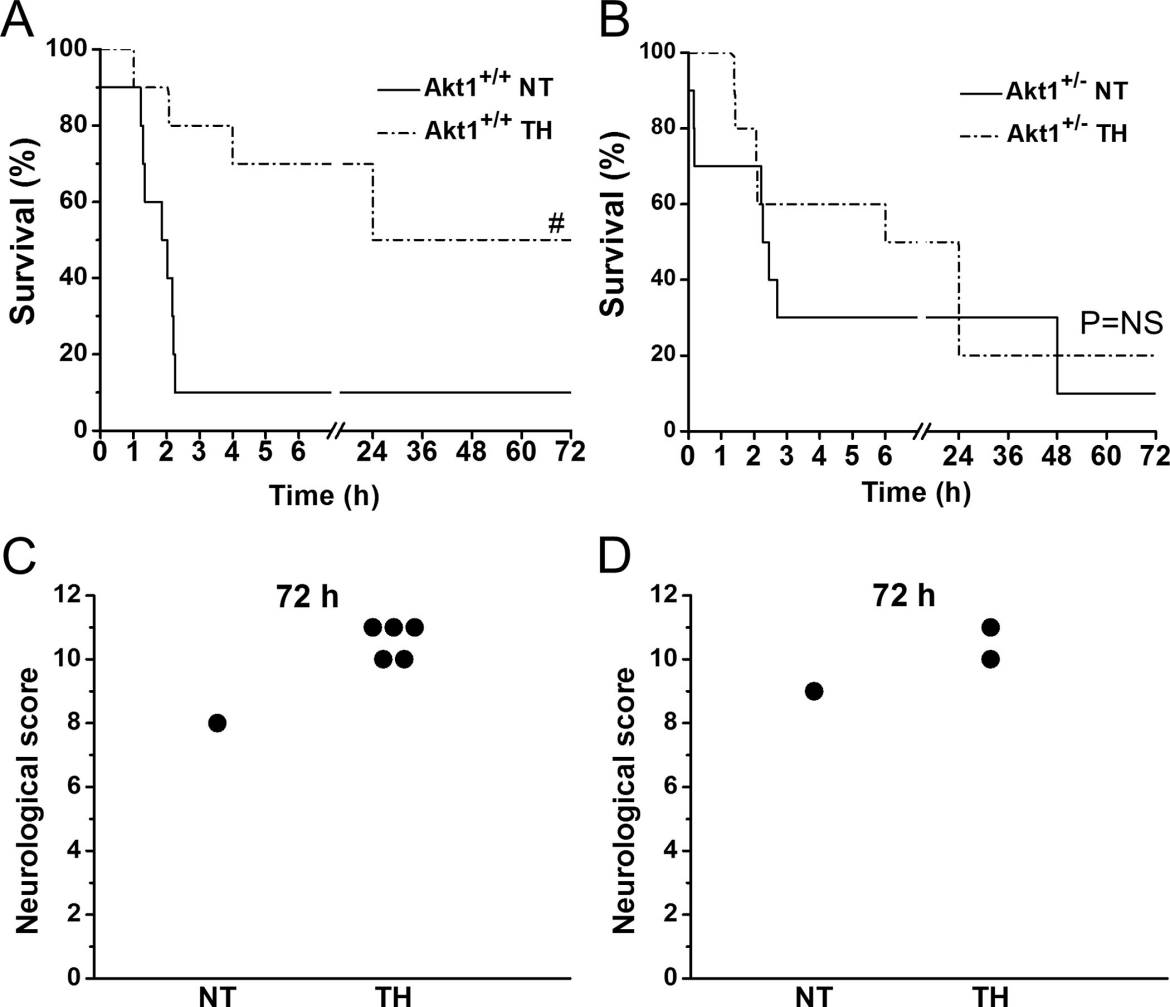
Effect of Akt1 on TH-induced neurological function and survival after SCA. *A*: Kaplan-Meier 72 h survival plot of NT and TH groups (n = 10 in each group, ^#^*p* < 0.05 between NT and TH groups) in Akt1^+/+^ mice. *B*: Kaplan-Meier 72 h survival plot of NT and TH groups (n = 10 in each group, *p* = NS) in Akt1^+/-^ mice. *C*: Assessment of Neurological function score (0 representing death of the animal and 12 reflecting a full neurological recovery) at R72h in Akt1^+/+^ mice. *D*: Assessment of neurological function score at R72h in Akt1^+/-^ mice.

Twenty mice each in Akt1^+/+^ and Akt1^+/-^ were randomized to NT and TH treatment group (n = 10 each group). Among Akt1^+/+^ mice, baseline characteristics including weight, temperature, heart rate, MAP and resuscitation parameters such as ROSC rate and time, were indistinguishable between the NT and TH group (Table 1). Compared to Akt1^+/+^ mice, Akt1^+/-^ mice were relatively smaller in size, but the characteristics at both baseline and resuscitation were similar as well in NT and TH treatment group. Among Akt1^+/+^ mice, 9 out of 10 mice in the NT group and 10 out of 10 mice in the TH group achieved ROSC, but only 1 mouse survived to 72 h in NT group while 5 out of 10 mice in TH group were alive at 72 h indicating TH confers protection against SCA injury (Fig 1A). For Akt1^+/-^ mice, 9 out of 10 mice in the NT group and 10 out of 10 mice achieved ROSC, similar to Akt1^+/+^ mice suggesting that TH had minimal effect on ROSC rates with this duration of cardiac arrest. 1 out of 9 mice in NT group survived to 72 h while 2 out of 10 mice in TH group lived to 72 h post-ROSC indicating a diminished TH protection in Akt1^+/-^ mice (Fig 1B). No difference in survival rate was observed between Akt^+/+^ and Akt^+/-^ with NT treatment (*p* = 0.22). However, even with the treatment of TH, survival rate does not differ between Akt^+/+^ and Akt^+/-^ mice (*p* = 0.21) further supporting that Akt1 mediates TH-induced protection.

**Table 1:** Characteristics at baseline and CPR period in Akt^+/+^NT, Akt^+/+^TH, Akt^+/-^NT and Akt^+/-^TH groups.

		Akt^+/+^NT (n = 10)	Akt^+/+^NT (n = 10)	*p* value	Akt^+/-^NT (n = 10)	Akt^+/-^NT (n = 10)	*p* value
**Baseline**							
	Body weight, g	28.0±1.4	28.5±2.0	0.21	26.5±2.4	25.9±3.4	0.66
	Heart rate, beats/min	304.3±38.7	308.9±67.1	0.82	305.0±65.3	304.7±40.8	1.03
	MAP (mmHg)	81.3±10.0	81.7±5.8	0.60	79.4±13.8	81.7±5.7	0.22
	P_ETCO2,_ mmHg	42.3±3.0	42.5±2.6	0.67	37.4±3.0	36.9±2.5	0.69
**CPR**	Heart rate, beats/min	458.8±59.5	412.6±41.4	0.06	463.0±52.6	420.0±71.0	0.21
	MAP (mmHg)	56.6±9.6	59.4±6.9	0.69	45.3±7.2	52.5±11.3	0.17
	P_ETCO2,_ mmHg	39.7±4.2	36.3±3.7	0.08	34.1±2.9	32.1±1.1	0.10
	Time to ROSC (s)	124.3±7.5	137.6±44.3	0.36	121.3±19.6	136.9±15.2	0.51
	ROSC, n(%)	9/10	10/10		9/10	10/10	

**Fig 2:**
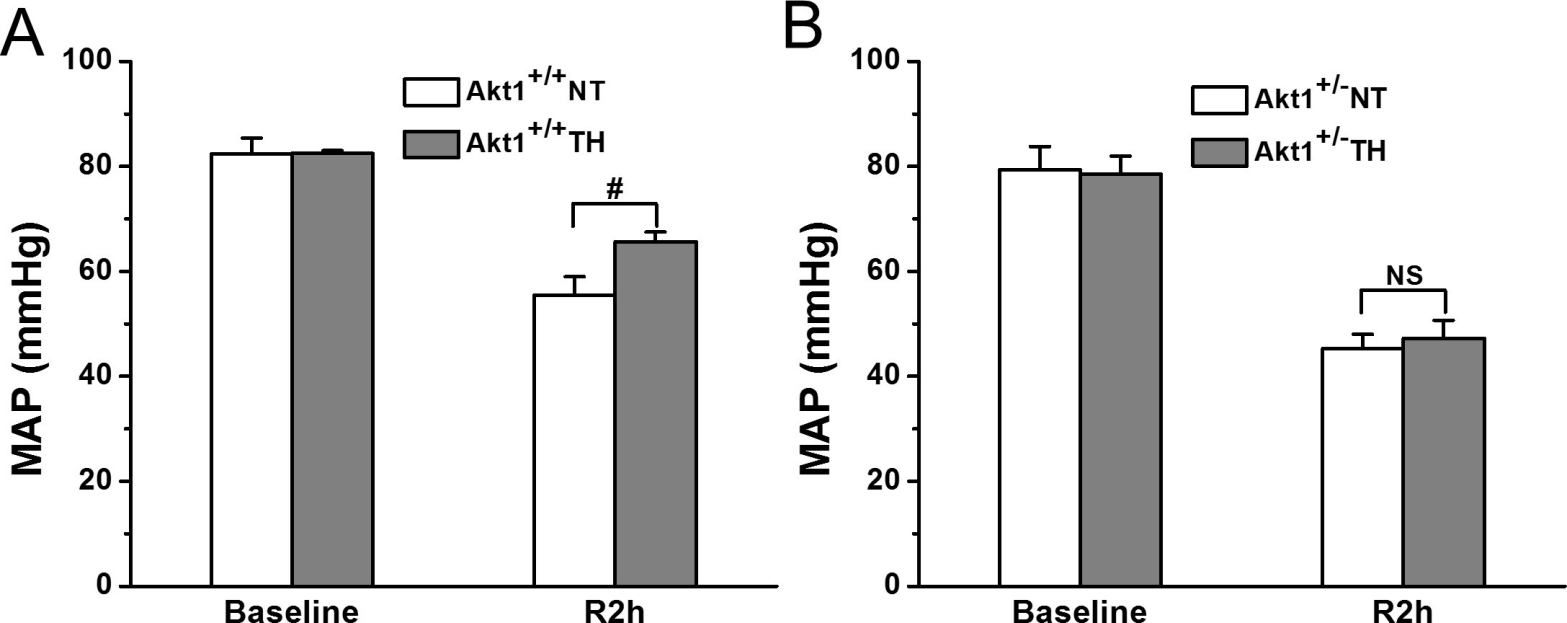
Effect of TH on hemodynamic performance after SCA in Akt1^+/+^ and Akt1^+/-^ mice. *A*: MAP at baseline and post-SCA R2h in NT and TH groups in Akt1^+/+^ mice was measured by a polyethylene (PE-10) catheter (n = 10, ^#^*p* < 0.05 between NT and TH groups). *B*: MAP at baseline and post-SCA R2h in NT and TH groups in Akt1^+/-^ mice was measured (n = 10, *p* = NS). Data presented are means ± SEM.

In Akt1^+/+^ mice, neurological score showed a trend of improvement about 40% from 8 in NT group to an average of 11.2 in TH group (Fig 1C). In Akt1^+/-^ mice, the number of survivor is too small to achieve a reasonable comparison on neurological scores (Fig 1D). An overall better recovery of MAP was noticed in Akt1^+/+^ compared to Akt1^+/-^ (Fig 2A and 2B). In Akt^+/+^ animals, MAP at 2h post ROSC was significantly increased in the TH versus NT group (Fig 2A). No MAP differences were noted in Akt^+/-^ animals (Fig 2B).

No difference was seen in the response to NT between Akt^+/+^ and Akt^+/-^ at 2 h after ROSC (*p* = 0.078) while TH-treated Akt^+/+^ mice had a better MAP recovery than Akt^+/-^ mice (*p* = 0.013).

### Akt1 deficiency decreased CPR TH-mediated Akt activation in both heart and brain, and downregulated phospholamban phosphorylation in heart

**Fig 3:**
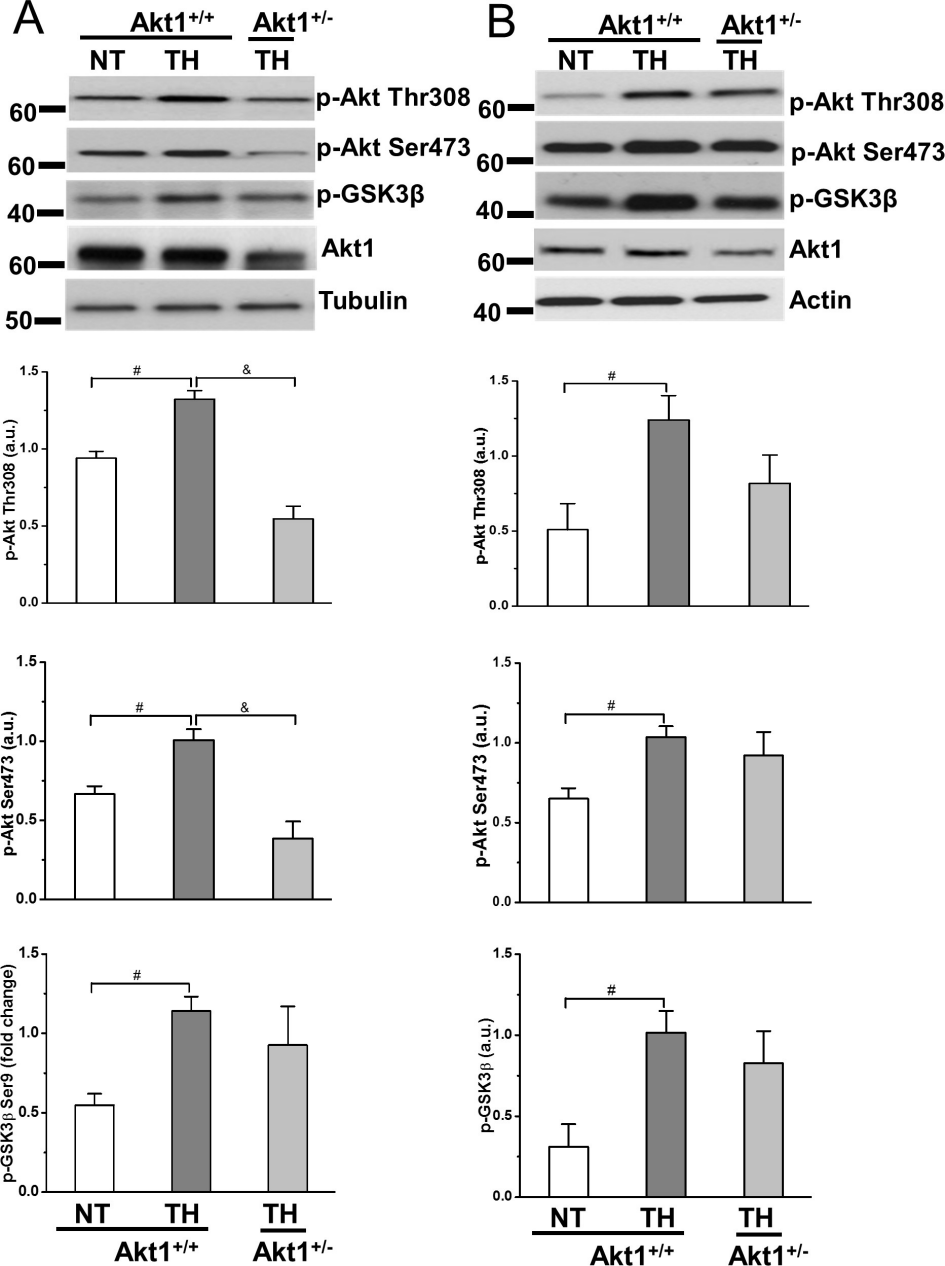
Phosphorylation of Akt and GSK3β was analyzed by Western blot at R30 in heart and brain of Akt1^+/+^ and Akt1^+/-^ mice with TH treatment. *A*: p-Akt Thr308, p-Akt Ser473 and p-GSK3β Ser9 at R30 in heart. α-tubulin was used as loading controls for heart. *B*: p-Akt Thr308, p-Akt Ser473 and p-GSK3β Ser9 at R30 in brain. β-actin was used as loading controls for brain. ^#^*p* < 0.05 between NT and TH groups of Akt1^+/+^; ^&^*p* < 0.05 between TH-treated groups of Akt1^+/+^ and Akt1^+/-^ mice. Data presented are means ± SEM of five mice.

Evidence of Akt activation was assessed using Akt phosphorylation (Thr308 and Ser473 sites) and GSK3β phosphorylation, using heart and brain tissue collected at R30 from mice treated with NT or TH in Akt1^+/+^ and compared with TH in Akt1^+/-^ mice. In the heart, TH increased Akt phosphorylation at both Thr308 and Ser473 in Akt1^+/+^ mice. Compared to Akt1^+/+^, TH-induced Akt phosphorylation was lower in Akt1^+/-^ mice despite the Akt1 expression was also notably lower in the heart of Akt1^+/-^ mice as expected (Fig 3A). The effect of TH on Akt phosphorylation and the role of Akt1^+/-^ on TH-mediated Akt phosphorylation in the brain are similar to the heart (Fig 3B). Further, GSK3β phosphorylation was increased with TH treatment in both heart and brain, confirming TH induced Akt activation. A similar trend of less TH-induced GSK3β phosphorylation in heart and brain was observed in Akt1^+/-^ mice compared to Akt1^+/+^ mice (Fig 3A and 3B).

**Fig 4:**
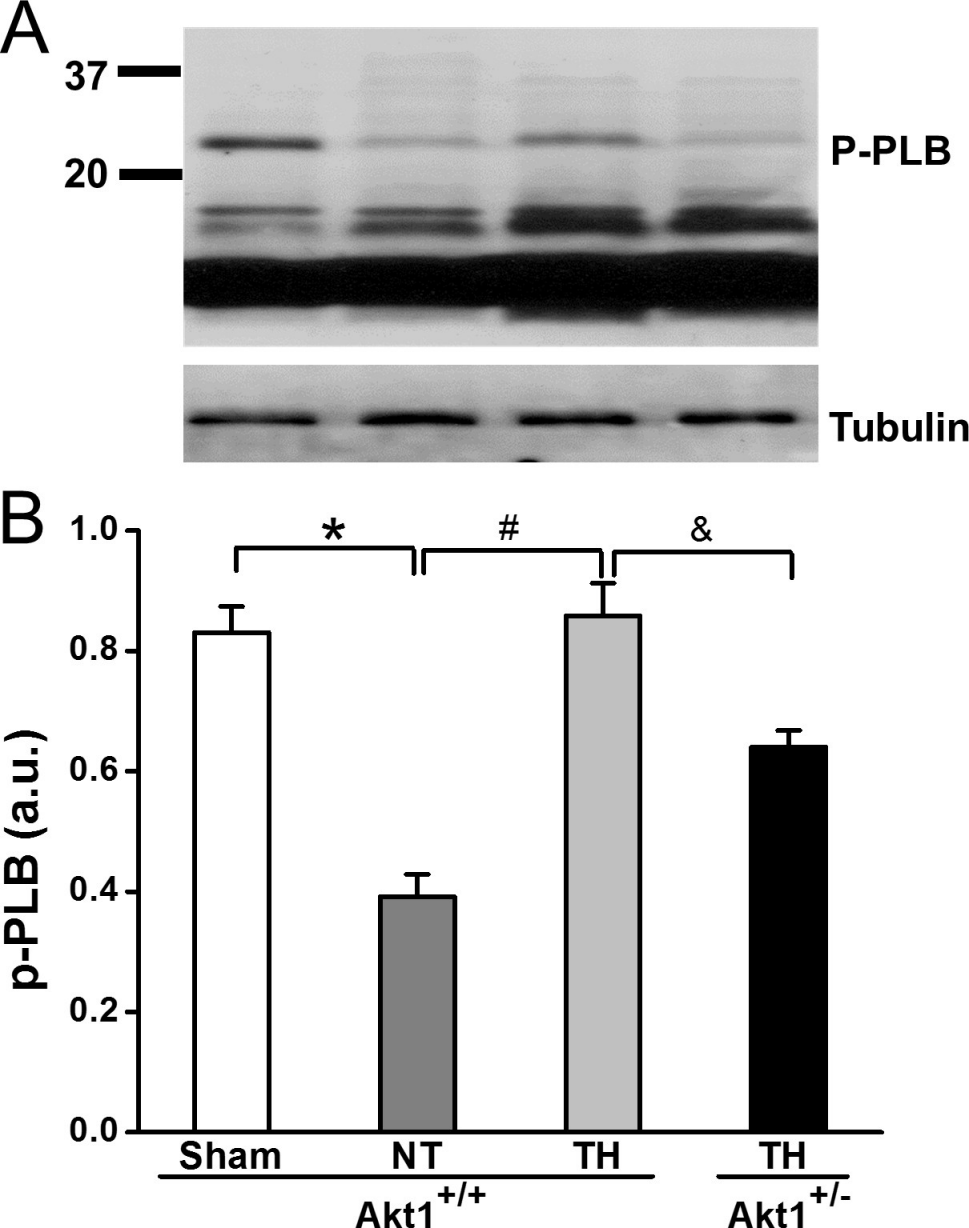
Phospholamban phosphorylation was analyzed by Western blot at R30 in hearts of Akt1^+/-^ and Akt1^+/-^ mice with TH treatment. Tubulin was used as a loading control. *p < 0.05 between sham and NT groups of Akt1^+/+^; ^#^*p* < 0.05 between NT and TH groups of Akt1^+/+^; ^&^*p* < 0.05 between TH-treated groups of Akt1^+/+^ and Akt1^+/-^ mice. Data presented are means ± SEM of four mice.

Phospholamban (PLB) is a small protein comprising 52 amino acids that can be directly phosphorylated at Thr17 by Akt1 [21]. Compared to sham, p-PLB was decreased in Akt1^+/+^ hearts at R30 after NT treatment while TH increased p-PLB. PLB phosphorylation in the hearts of Akt1^+/-^ mice treated with TH was significantly lower than that in Akt1^+/+^ mice. (Fig 4).

### Akt1 deficiency reduced CPR TH-enhanced pyruvate dehydrogenase activation

**Fig 5:**
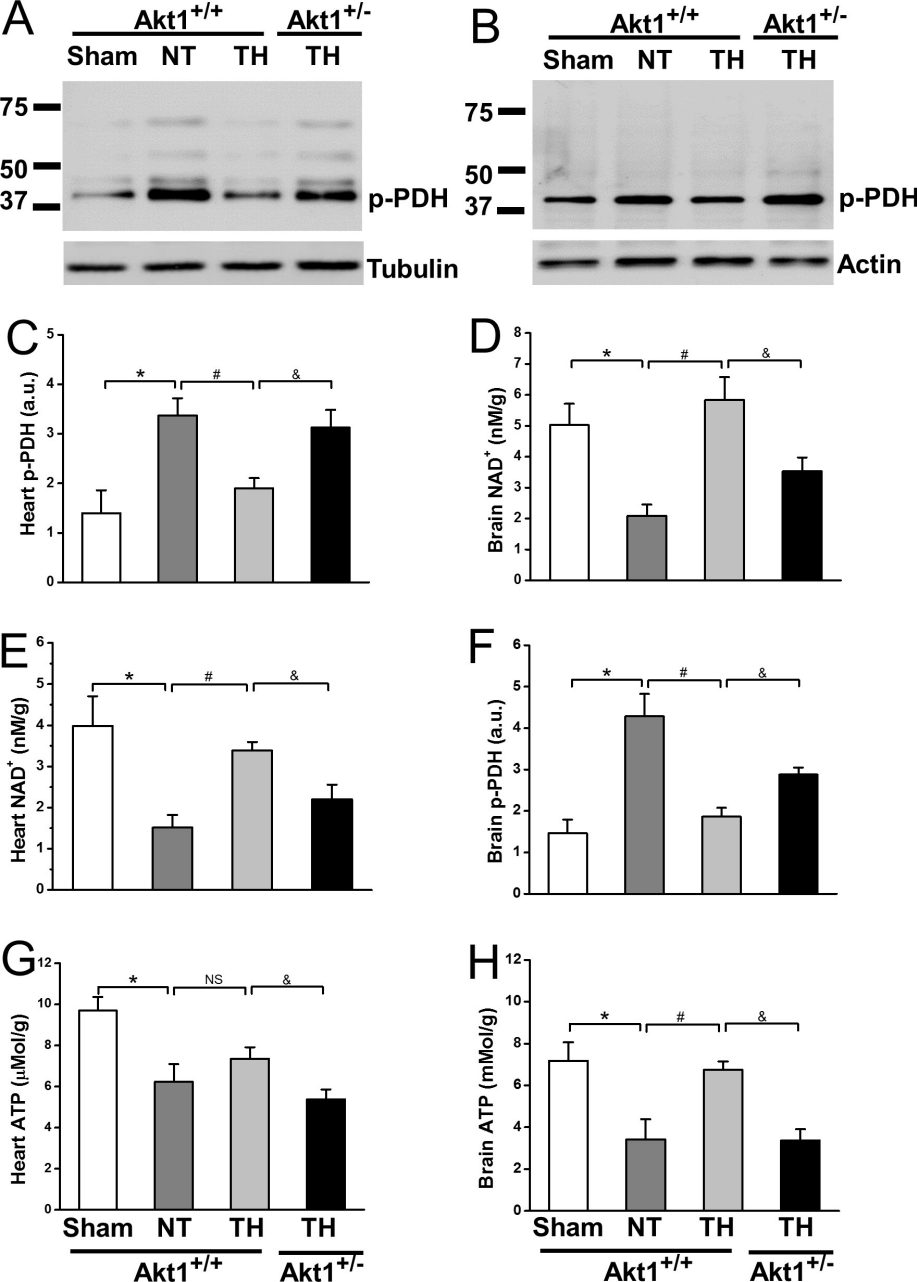
**PDH Phosphorylation, and NAD and ATP contents were measured at R30 in heart (A, C, E, G) and brain (B, D, F, H) in Akt1+/+ and Akt1+/- mice treated with TH**. *A*, *C*: PDH Phosphorylation at R30 in heart. α-tubulin was used as loading controls. *B*, *D*: PDH Phosphorylation at R30 in brain. β-actin was used as loading controls for brain. *E*: NAD^+^ contents at R30 in heart. *F*: NAD^+^ contents at R30 in brain. *G*: ATP contents at R30 in heart. *H*: ATP contents at R30 in brain. *p < 0.05 between sham and NT groups of Akt1^+/+^ mice; ^#^*p* < 0.05 between NT and TH groups of Akt1^+/+^ mice; ^&^*p* < 0.05 between TH-treated groups of Akt1^+/+^ and Akt1^+/-^ mice. Data presented are means ± SEM of five mice.

Pyruvate dehydrogenase (PDH), a rate limiting enzyme for the conversion of pyruvate derived from glycolysis to acetyl-CoA for TCA cycle activity and ATP generation [22]. Decreased PDH phosphorylation results in increased PDH activity [23]. Compared to sham, NT increased PDH E1-α subunit phosphorylation (Ser293) at R30 in heart (Fig 5A and 5C) and brain tissues (Fig 5B and 5D) of Akt1^+/+^ mice. TH prevented PDH phosphorylation in both tissues. PDH phosphorylation in the heart of Akt1^+/-^ mice treated with TH was higher than that in Akt1^+/+^ mice and this was also true for brain suggesting that Akt1 regulates TH-enhanced PDH activity. Consistent with increased PDH activity, bioenergetic outcomes including NAD^+^ and ATP in the heart and brain were reduced by NT and improved by TH. TH increased NAD^+^ levels significantly in the heart (Fig 5E) and brain (Fig 5F) of Akt1^+/+^ mice, an effect abrogated in Akt1^+/-^ mice. Regarding ATP concentrations, TH did not increase ATP in the heart as much as in the brain of Akt1^+/+^ mice (Fig 5G and 5H), However, ATP levels were significantly lower in both the heart and brain of Akt1^+/-^ compared to Akt1^+/+^ mice with TH treatment.

### Akt1 deficiency partially reversed CPR TH-suppressed NF-κB activation

**Fig 6:**
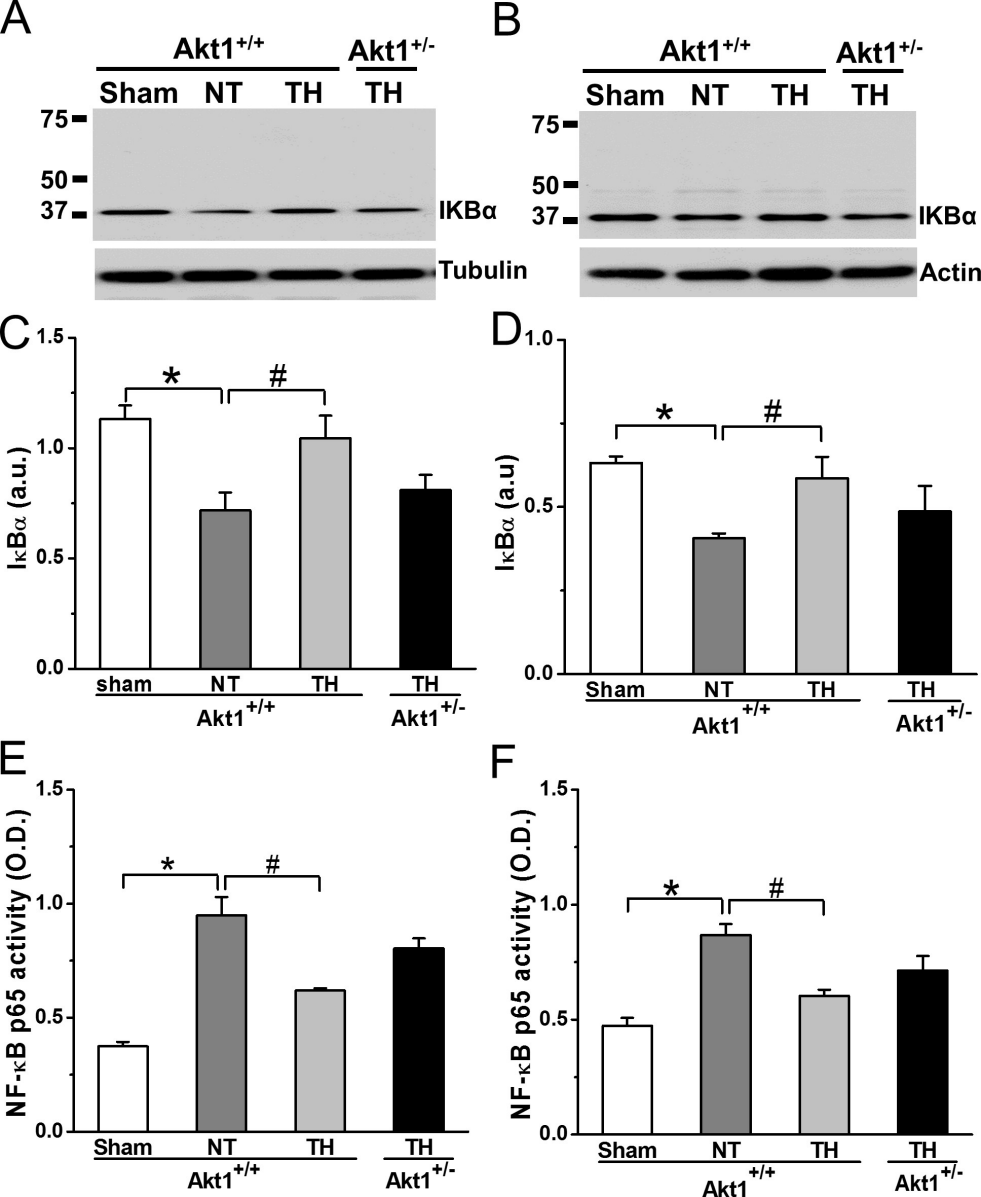
IκBα degradation and NF-κB activity at R30 in heart and brain of Akt1^+/+^ and Akt1^+/-^ with TH treatment. *A*, *C*: IκBα and NF-κB at R30 in heart. α-tubulin was used as loading controls for heart. *B*, *D*: IκBα and NF-κB at R30 in brain. β-actin was used as loading controls for brain. *p < 0.05 between sham and NT groups of Akt1^+/+^ mice; ^#^*p* < 0.05 between NT and TH groups of Akt1^+/+^ mice. Data presented are means ± SEM of five mice.

Given the recognized role of inflammation in cardiac arrest injury, we measured markers of inflammation that may be related to Akt1 downstream signaling. NF-κB is activated by proinflammatory cytokines, endogenous ligands and ROS generated following I/R and regulates numerous genes in survival and inflammatory pathways in both heart and brain [24, 25]. IκBα degradation is indicative of NF-κB activation and was assessed in both heart and brain tissues of Akt1^+/+^ and Akt1^+/-^ mice collected at R30. Compared to sham, IκBα degradation and subsequent NF-κB (p50/p65) nuclear binding activity was increased with NT treatment in both heart (Fig 6A, 6C and 6E) and brain (Fig 6B, 6D and 6F) of Akt1^+/+^ mice. TH preserved IκBα protein and inhibited NF-κB activation in Akt1^+/+^ while these effects were partially reversed in Akt1^+/-^ mice treated with TH. A trend of increase in IκBα degradation and NF-κB activation were seen in both tissues of Akt1^+/-^ mice treated with TH compared to those of Akt1^+/+^ mice suggesting that NF-κB activation may be in part regulated by Akt1.

### CPR cooling reduced a conventional blood marker lactate and a novel blood marker PBEF, and Akt1 deficiency abolished these effects

**Fig 7:**
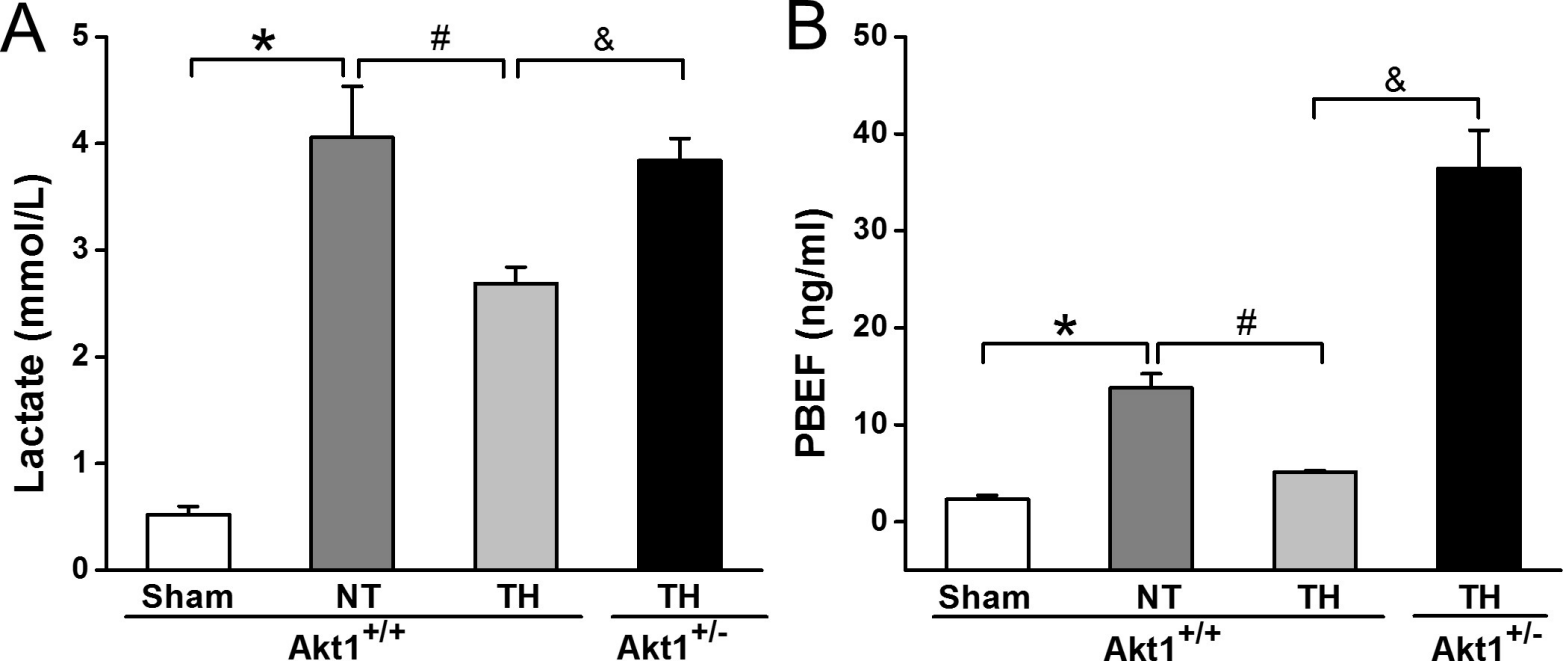
Plasma lactate and PBEF concentrations were assessed at R30 in Akt1^+/+^ and Akt1^+/-^ mice with TH treatment TH. *A*: Plasma PBEF. *B*: Plasma lactate. *p < 0.05 between sham and NT groups of Akt1^+/+^ mice; ^#^*p* < 0.05 between NT and TH groups of Akt1^+/+^ mice; ^&^*p* < 0.05 between TH-treated groups of Akt1^+/+^ and Akt1^+/-^ mice. Data presented are means ± SEM of five mice.

Lactate, the conventional metabolic marker in the blood related to cardiac arrest injury, was increased from 0.52 ± 0.08 mmol/L in sham to 4.06 ± 0.48 mmol/L at R30 with NT in Akt1^+/+^ mice. TH reduced lactate level to 2.69 ± 0.15 mmol/L in Akt1^+/+^ mice, but not in Akt1^+/-^ mice (3.84 ± 0.21 mmol/L) (Fig 7A). PBEF, also known as extracellular nicotinamide phosphoribosyltransferase (Nampt) and visfatin that regulates both metabolism and inflammation, was measured at R30 [26]. As depicted in Fig 7B, plasma PBEF was markedly increased approximately 6 fold with NT (2.30 ± 0.42 ng/ml for sham vs. 13.8 ± 1.45 ng/ml for NT) and reduced by TH (5.12 ± 0.17 ng/ml). It was notably increased in Akt1^+/-^ mice despite TH treatment (36.43 ± 3.92 ng/ml in Akt1^+/-^ vs. 5.12 ± 0.17 ng/ml in Akt1^+/+^), more than the changes of lactate in Akt1^+/-^ mice treated with TH.

## Discussion

The present study builds upon our prior work suggesting that TH protection of cardiac function and survival after ischemia/reperfusion injury as seen in SCA is associated with Akt activation [14, 27]. This study helps identify Akt1-mediated CPR TH mechanisms of protection related to contractile function, metabolic recovery and inflammatory responses during early resuscitation.

The great majority of studies of TH for SCA have focused on its neurological protection. Some prior work in both clinical and animal studies support the notion that when TH is induced immediately after ROSC [11, 13] or during CPR, it affects cardiac protection as well as improves neurologic outcomes. A heart-brain connection in TH protection likely exists. The recovery of MAP within the first hours after CPR has been linked to later neurologically intact survival due to improved recovery of brain perfusion [8, 28]. The results of the current work suggest that Akt1, the only Akt isoform abundant in both heart and brain, appears to improve cardiac contraction through upregulating phospholamban phosphorylation within 30 min after CPR, allowing for early, improved brain perfusion. Contractile dysfunction is a hallmark of SCA injury and is attributed to both impairment of PDH activity and Ca^2+^ related contractile function. Phospholamban is one of the key proteins modulating Ca^2+^ homeostasis via binding to SERCA2a and inhibiting it when nonphosphorylated or dephosphorylated [29]. Phospholamban in hearts from NT mice was dephosphorylated compared to sham mice indicating the inhibition of SERCA2a function within 30 min after CPR, and consistent with Ca^2+^ overload and impairment of contractile function. TH treatment enhanced phospholamban phosphorylation leading to enhanced SERCA-related Ca^2+^ uptake by sarcoplasmic reticulum with stronger excitation/contraction coupling of cardiac cells and increased MAP at 30 min after CPR. While novel to the context of cardiac arrest and CPR TH protection, the current study is supported by the work of others showing that hypothermia possesses robust inotropic effect that may involve other mechanisms including increased Ca^2+^ sensitivity of myofilament proteins and improved Ca^2+^-activated force generation [30–33]. Specific to phospholamban, a prior study showed that rabbit hearts exposed to 4⁰C for 72 hours significantly increased its phosphorylation while decreasing the expression [34]. The present study is one of the first reports suggesting that CPR TH of only a few degrees can rapidly upregulate phospholamban phosphorylation and contractile function within 30 minutes. These effects were diminished in Akt1^+/-^ mice compared to Akt1^+/+^ mice suggesting a role for Akt1 in mediating the positive inotropic effects of TH. This finding is supported by the study demonstrating that Akt activation directly increases PLB phosphorylation at Thr17 site [21].

While many reviews of TH protection in the context of SCA suggest a mechanism of decreased metabolism, our results suggest that TH actually enhances glucose utilization via increased PDH activity. This is consistent with other reports indicating a critical role for PDH impairment in both cardiac stunning and neurological recovery from ischemic injury [6, 7]. The present study demonstrates that TH decreased PDH phosphorylation (i.e. increased PDH activity) in Akt1^+/+^ mice compared to NT mice. This is supported by a previous report that TH enhances PDH activity following cardiac arrest [35]. Such increased PDH activity may facilitate the transportation of pyruvate generated from enhanced glycolysis into mitochondria for oxidation, with conversion of NADH to NAD^+^ and increased ATP production leading to reduced conversion of pyruvate to lactate to ease post-SCA acidosis and enhanced energy replenishment to promote ion exchange and correction of contractile dysfunction [36, 37]. To investigate the role that Akt1 plays in TH-enhanced metabolic recovery, we compared the PDH phosphorylation in the heart and brain of both Akt1^+/+^ and Akt1^+/-^ mice treated with TH and found that PDH phosphorylation was increased in Akt1^+/-^ mice despite TH treatment suggesting that Akt1 is critical for PDH phosphorylation. Previous studies suggested that Akt1 regulates mainly cell growth while Akt2 is the main isoform involved in glucose metabolism, However, others reported that Akt1 also play a role in glucose metabolism [38, 39]. PDH is a critical enzyme that regulates glucose oxidation, an important aspect of glucose metabolism, and its activity is controlled by a family of four PDH kinase (PDK1-PDK4) [40, 41]. Both Akt1 and Akt2 were demonstrated to phosphorylate PDK1 [42], and PDK4 expression was markedly inhibited by infection with shRNA directed against Akt1 [43]. In the present study, PDH activity was significantly affected in Akt1+/- mice providing further support for an important role of Akt1 in regulating glucose metabolism in addition to its role in modulating cell growth. Moreover, lower levels of ATP in the heart than the brain were observed at R30 in both the present study and our previous study using a PTEN inhibitor VO-OHpic [18]. We postulate that a large amount of ATP was utilized to support the improvements in heart contraction and function during early resuscitation. Furthermore, the present study demonstrates that TH trended to inhibit IκBα degradation and NF-κB nuclear translocation in addition to others reported mechanisms including reduction of neutrophil activation and ROS generation during resuscitation [12, 44], and decrease of enzymatic activity of IKK-γ, an activator of NF-κB [45–47]. Beside these effects, TH can also decrease membrane penetration of NF-κB by altering the biophysical properties of membrane lipids to decrease fluidity [48, 49]. The Akt1-NF-κB regulation axis was suggested in a previous report that L Protein of Parainfluenza Virus 5 triggered Akt1-Dependent Activation of NF-κB [50]. Akt1 partially reversed TH effects in the present study indicates that other NF-κB regulators such as PKC and p38 may be involved [51, 52]. TH-induced metabolic recovery and inflammation suppression via upregulating PDH activation and downregulating NF-κB activity, respectively, further improve myocardial contractile function and brain perfusion, ultimately neurologically intact SCA survival.

Our present work highlights a potentially important role for Akt1-related suppression of plasma PBEF concentrations in CPR TH protection. It is remarkable that PBEF concentrations were not only reduced by CPR TH, but greatly increased in Akt1 deficient mice despite TH treatment. Since PBEF release from tissue into plasma is linked to both inflammatory pathways such as NF-κB and metabolic pathway such as tissue NAD^+^ depletion, it is conceivable that the cumulative effect of Akt1 in affecting both of these aspects resulted in this outcome. More work is needed to understand the role of PBEF in CPR TH protection.

### Limitations

There are several limitations in the present study. Given the lethal phenotype of complete Akt1 knockout mice, it was only possible to study Akt1 haplo-deficient animals. However, given that TH likely affects many targets, such as Akt, AMP-activated protein kinase, Dynamin Related Protein 1 (Drp1) [18, 53, 54], it is notable that even partial deficiency of Akt1 resulted in abrogation of TH protection and critical aspects of contractile, metabolic and inflammatory regulation. SCA is a multi-organ disease of ischemia/reperfusion, it is likely that other organs in addition to heart and brain were affected. We chose to focus on the two critical organs reported to be affected most by CPR TH. Akt1 likely has other downstream targets that may also mediate TH protection, such as Akt1 on regulation of NOS3 [55]. We have reported a substantial increase in post-cardiac arrest injury seen in NOS3^-/-^ mice [56]. Our goal was not to study an exhaustive list of Akt1 targets, but to show that Akt1 plays an important role in TH CPR protection on some of the important aspects related to cardiac stunning, recovery of glucose oxidation and attenuation of inflammation. We also acknowledged that the effects of NT on these targets in Akt1^+/-^ mice were not assessed which limit the understanding of the role of TH on these aspects in these mice. However, our intent was to explore the role of Akt1 on TH-induced recovery of multiple targets. In addition, it is quite possible that other Akt isoforms play a role in TH protection, but it was beyond the scope of work to explore other isoforms. The activity of PDH was not measured. However, the concept of using PDH phosphorylation to reflect its activity has been well accepted. Finally, mechanisms by which Akt1 regulate plasma PBEF concentrations warrant future investigation.

### Clinical implications

Active cooling during CPR is very protective against SCA injury, but difficult to implement clinically. Our current study investigated the mechanisms and targets of Akt1-mediated cooling protection. This may provide insights into potential pharmacological therapeutics that would target Akt1 or its specific regulators during CPR to maximize TH effects to improve SCA survival without the need for physical cooling.

## Conclusions

CPR TH can exert powerful protection against SCA injury despite cooling by just a few degrees in temperature and maintained for only an hour after CPR. However, it is difficult to implement in the clinical setting due to the challenges of heat transfer during conditions of low cardiac output. This study reveals potential mechanisms of Akt1-mediated TH protection including increased phospholamban phosphorylation and contractile function, enhanced metabolism through increased PDH activation and glucose utilization for bioenergetics recovery, and downregulated IκBα degradation and NF-κB activity, and related metabolic and inflammatory markers lactate and PBEF. Together, these results suggests several potential targets for new CPR medications.
